# 4-Chloro-2,5-dimethyl­quinoline

**DOI:** 10.1107/S1600536810025419

**Published:** 2010-07-14

**Authors:** K. Prabha, K. N. Vennila, K. J. Rajendra Prasad, D. Velmurugan

**Affiliations:** a Department of Chemistry, Bharathiar University, Coimbatore 641 046, India; b Centre of Advanced Study in Crystallography and Biophysics, University of Madras, Guindy Campus, Chennai 600 025, India

## Abstract

Mol­ecules of the title compound, C_11_H_10_ClN, are essentially planar (r.m.s. deviation for all non-H atoms = 0.009 Å) and are stacked along the *a* axis with the centroids of the benzene and pyridine rings alternately separated by 3.649 (1) and 3.778 (1) Å.

## Related literature

For the biological activity of quinoline derivatives, see: Miyamoto *et al.* (1995 ▶); Milner *et al.* (2010 ▶); Li *et al.* (2008 ▶); Musiola *et al.* (2006 ▶); Muthumani *et al.* (2010 ▶). For related chloro­quinoline structures, see: Rizvi *et al.* (2008 ▶); Bureau *et al.* (1999 ▶); de Souza *et al.* (2010 ▶); Yathirajan *et al.* (2007 ▶). 
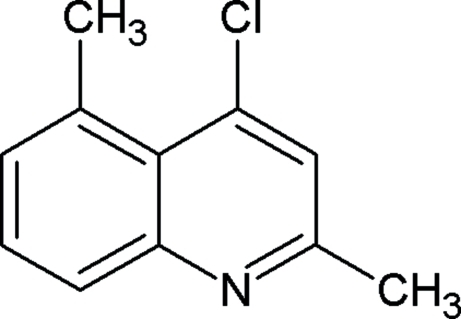


## Experimental

### 

#### Crystal data

C_11_H_10_ClN
*M* *_r_* = 190.66
Monoclinic, 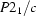

*a* = 6.9534 (9) Å
*b* = 13.0762 (14) Å
*c* = 10.4306 (11) Å
β = 99.239 (8)°
*V* = 936.09 (19) Å^3^
*Z* = 4
Mo *K*α radiation
μ = 0.35 mm^−1^
*T* = 293 K
0.27 × 0.26 × 0.22 mm

#### Data collection

Bruker SMART APEXII area-detector diffractometer
Absorption correction: multi-scan (*SADABS*; Bruker, 2008 ▶) *T*_min_ = 0.909, *T*_max_ = 0.925
8798 measured reflections
2345 independent reflections
1502 reflections with *I* > 2σ(*I*)
*R*_int_ = 0.027

#### Refinement

*R*[*F* ^2^ > 2σ(*F* ^2^)] = 0.050
*wR*(*F* ^2^) = 0.147
*S* = 1.05
2345 reflections
120 parameters
H-atom parameters constrained
Δρ_max_ = 0.23 e Å^−3^
Δρ_min_ = −0.21 e Å^−3^

### 

Data collection: *APEX2* (Bruker, 2008 ▶); cell refinement: *SAINT* (Bruker, 2008 ▶); data reduction: *SAINT*; program(s) used to solve structure: *SHELXS97* (Sheldrick, 2008 ▶); program(s) used to refine structure: *SHELXL97* (Sheldrick, 2008 ▶); molecular graphics: *ORTEP-3* (Farrugia, 1997 ▶); software used to prepare material for publication: *SHELXL97* and *PLATON* (Spek, 2009 ▶).

## Supplementary Material

Crystal structure: contains datablocks global, I. DOI: 10.1107/S1600536810025419/ci5114sup1.cif

Structure factors: contains datablocks I. DOI: 10.1107/S1600536810025419/ci5114Isup2.hkl

Additional supplementary materials:  crystallographic information; 3D view; checkCIF report

## Figures and Tables

**Fig. 1.**
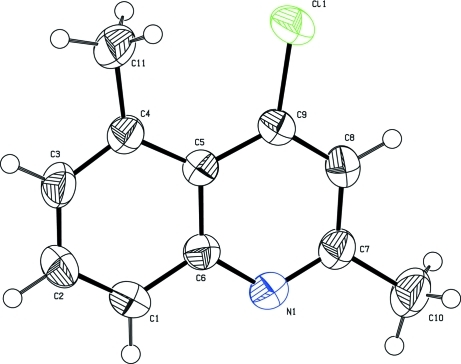
The molecular structure of the title compound. Displacement ellipsoids are drawn at the 50% probability level.

**Fig. 2.**
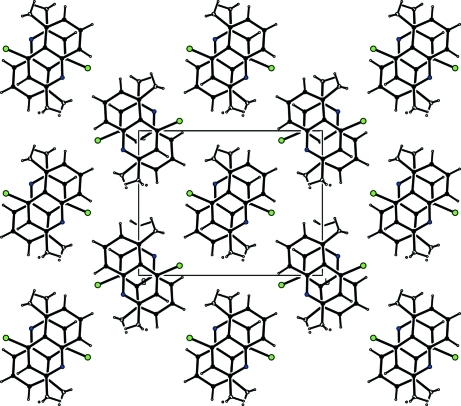
The crystal packing of the title compound, viewed down the a axis.

## supplementary crystallographic information

### Comment

This paper presents the first crystal structure of *meta* isomer of chloro-quinoline derivatives. It was reported earlier that the introduction of methyl group at the 5th position of quinoline nucleus enhanced characteristically the antibacterial activity against Gram-positive bacteria, including Streptococcus pneumonia, which is a major pathogen in the respiratory tract infection (Miyamoto *et al.*, 1995). Also quinoline derivatives are well known to have many biological activities such as antimalarial (Milner *et al.*, 2010), inhibition of melanogenesis (Li *et al.*, 2008), antifungal (Musiola *et al.*, 2006), antibacterial activities *etc.* (Muthumani *et al.*, 2010).

The non-hydrogen atoms of the title molecule are essentially coplanar (r.m.s. deviation 0.009 Å). The molecules are stacked along the *a* axis with the centroids of quinoline ring systems alternately separated by 3.649 (1) Å and 3.778 (1) Å (Fig.2).

### Experimental

Ethylacetoacetate (0.25 mol) and *m*-toluidine (0.25 mol) were mixed. 5–10 drops of dilute hydrochloroacid (1:1) was added, the mixture was shaken well and kept inside a vacuum desiccator over concentrated sulfuric acid for 2 d. A deep yellow oily liquid, (*E*)-Ethyl-3-(*m*-tolylamino)but-2-enoate, was formed. It was dried over anhydrous sodium sulfate and was added dropwise from a dropping funnel to diphenyl ether (50 ml) kept at reflux in a two necked flask, one fitted with the dropping funnel and the other with an air condenser to distill off the ethanol formed during the reaction. After the addition, the refluxing was continued for further 10 min and the contents were cooled. 50 ml of petroleum ether was added and the precipitated solid was collected, washed with petroleum ether, dried and recrystallized from ethanol to give (E)-ethyl-3-(m-tolylamino)but-2-enoate as a crystalline white powder.

Phosphorous oxy chloride (100 ml) was added to 2,5-dimethylquinolin-4 (1*H*)-one (0.1 mol) and kept on a water bath for about 1 h and poured into ice water and neutralized with saturated sodium carbonate solution. The formed precipitate was filtered, dried, purified using silica gel column chromatography and eluted with petrolelum ether (100%) to get a white solid. It was recrystallized using methanol.

### Refinement

H atoms were positioned geometrically [C–H = 0.93 or 0.96 Å] and were allowed to ride on their parent atoms, with *U*_iso_ = 1.5*U*_eq_(C) for methyl H and 1.2*U*_eq_(C) for other H atoms.

### Crystal data

**Table:**

C_11_H_10_ClN	*F*(000) = 400
*M**_r_* = 190.66	*D*_x_ = 1.353 Mg m^−^^3^
Monoclinic, *P*2_1_/*c*	Mo *K*α radiation, λ = 0.71073 Å
Hall symbol: -P 2ybc	Cell parameters from 2345 reflections
*a* = 6.9534 (9) Å	θ = 2.5–28.5°
*b* = 13.0762 (14) Å	µ = 0.35 mm^−^^1^
*c* = 10.4306 (11) Å	*T* = 293 K
β = 99.239 (8)°	Block, colourless
*V* = 936.09 (19) Å^3^	0.27 × 0.26 × 0.22 mm
*Z* = 4	

### Data collection

**Table:**

Bruker SMART APEXII area-detector diffractometer	2345 independent reflections
Radiation source: fine-focus sealed tube	1502 reflections with *I* > 2σ(*I*)
graphite	*R*_int_ = 0.027
ω and φ scans	θ_max_ = 28.5°, θ_min_ = 2.5°
Absorption correction: multi-scan (*SADABS*; Bruker, 2008)	*h* = −9→9
*T*_min_ = 0.909, *T*_max_ = 0.925	*k* = −17→12
8798 measured reflections	*l* = −13→10

### Refinement

**Table:**

Refinement on *F*^2^	Primary atom site location: structure-invariant direct methods
Least-squares matrix: full	Secondary atom site location: difference Fourier map
*R*[*F*^2^ > 2σ(*F*^2^)] = 0.050	Hydrogen site location: inferred from neighbouring sites
*wR*(*F*^2^) = 0.147	H-atom parameters constrained
*S* = 1.05	*w* = 1/[σ^2^(*F*_o_^2^) + (0.0468*P*)^2^ + 0.6973*P*] where *P* = (*F*_o_^2^ + 2*F*_c_^2^)/3
2345 reflections	(Δ/σ)_max_ = 0.007
120 parameters	Δρ_max_ = 0.23 e Å^−^^3^
0 restraints	Δρ_min_ = −0.21 e Å^−^^3^

### Special details

**Table:**

Geometry. All e.s.d.'s (except the e.s.d. in the dihedral angle between two l.s. planes) are estimated using the full covariance matrix. The cell e.s.d.'s are taken into account individually in the estimation of e.s.d.'s in distances, angles and torsion angles; correlations between e.s.d.'s in cell parameters are only used when they are defined by crystal symmetry. An approximate (isotropic) treatment of cell e.s.d.'s is used for estimating e.s.d.'s involving l.s. planes.
Refinement. Refinement of *F*^2^ against ALL reflections. The weighted *R*-factor *wR* and goodness of fit *S* are based on *F*^2^, conventional *R*-factors *R* are based on *F*, with *F* set to zero for negative *F*^2^. The threshold expression of *F*^2^ > σ(*F*^2^) is used only for calculating *R*-factors(gt) *etc*. and is not relevant to the choice of reflections for refinement. *R*-factors based on *F*^2^ are statistically about twice as large as those based on *F*, and *R*- factors based on ALL data will be even larger.

### Fractional atomic coordinates and isotropic or equivalent isotropic displacement parameters (Å^2^)

**Table:**

	*x*	*y*	*z*	*U*_iso_*/*U*_eq_	
Cl1	0.27505 (13)	0.22264 (5)	0.06695 (7)	0.0680 (3)	
N1	0.2186 (3)	−0.08447 (15)	−0.13203 (18)	0.0455 (5)	
C5	0.2644 (3)	0.01002 (16)	0.0758 (2)	0.0372 (5)	
C8	0.2282 (4)	0.09687 (19)	−0.1312 (2)	0.0461 (6)	
H8	0.2218	0.1571	−0.1791	0.055*	
C9	0.2550 (4)	0.10065 (17)	0.0003 (2)	0.0410 (5)	
C1	0.2513 (4)	−0.17776 (18)	0.0632 (2)	0.0485 (6)	
H1	0.2379	−0.2374	0.0140	0.058*	
C6	0.2455 (3)	−0.08204 (17)	0.0005 (2)	0.0383 (5)	
C3	0.2963 (4)	−0.0945 (2)	0.2683 (2)	0.0504 (6)	
H3	0.3140	−0.1002	0.3583	0.060*	
C10	0.1806 (5)	−0.0015 (2)	−0.3412 (2)	0.0622 (8)	
H10A	0.1524	−0.0705	−0.3697	0.093*	
H10B	0.2969	0.0213	−0.3712	0.093*	
H10C	0.0738	0.0420	−0.3760	0.093*	
C4	0.2913 (4)	0.00173 (18)	0.2147 (2)	0.0426 (5)	
C7	0.2103 (4)	0.00246 (19)	−0.1951 (2)	0.0443 (6)	
C11	0.3095 (5)	0.0919 (2)	0.3066 (2)	0.0629 (8)	
H11A	0.3289	0.0677	0.3946	0.094*	
H11B	0.1926	0.1321	0.2905	0.094*	
H11C	0.4186	0.1332	0.2931	0.094*	
C2	0.2761 (4)	−0.18365 (19)	0.1949 (2)	0.0529 (7)	
H2	0.2796	−0.2470	0.2356	0.064*	

### Atomic displacement parameters (Å^2^)

**Table:**

	*U*^11^	*U*^22^	*U*^33^	*U*^12^	*U*^13^	*U*^23^
Cl1	0.1124 (7)	0.0347 (3)	0.0570 (4)	−0.0066 (4)	0.0141 (4)	−0.0037 (3)
N1	0.0560 (13)	0.0426 (11)	0.0382 (10)	0.0017 (9)	0.0092 (9)	−0.0031 (8)
C5	0.0383 (12)	0.0372 (11)	0.0365 (11)	0.0000 (10)	0.0076 (9)	−0.0003 (9)
C8	0.0556 (16)	0.0430 (13)	0.0403 (13)	−0.0010 (11)	0.0094 (11)	0.0077 (10)
C9	0.0480 (14)	0.0338 (11)	0.0417 (12)	−0.0024 (10)	0.0082 (10)	−0.0006 (9)
C1	0.0601 (17)	0.0341 (11)	0.0520 (14)	0.0037 (11)	0.0106 (12)	0.0004 (10)
C6	0.0406 (13)	0.0375 (11)	0.0370 (11)	0.0026 (9)	0.0068 (9)	−0.0004 (9)
C3	0.0599 (16)	0.0566 (15)	0.0349 (12)	0.0031 (13)	0.0086 (11)	0.0099 (11)
C10	0.077 (2)	0.0737 (19)	0.0356 (13)	−0.0033 (16)	0.0085 (13)	0.0012 (12)
C4	0.0484 (14)	0.0439 (12)	0.0360 (12)	−0.0023 (10)	0.0085 (10)	−0.0012 (9)
C7	0.0497 (15)	0.0483 (13)	0.0349 (12)	−0.0005 (11)	0.0070 (10)	0.0008 (10)
C11	0.094 (2)	0.0591 (16)	0.0353 (13)	−0.0126 (15)	0.0096 (13)	−0.0094 (12)
C2	0.0654 (18)	0.0398 (12)	0.0545 (15)	0.0082 (12)	0.0121 (13)	0.0137 (11)

### Geometric parameters (Å, °)

**Table:**

Cl1—C9	1.737 (2)	C3—C4	1.374 (3)
N1—C7	1.310 (3)	C3—C2	1.390 (4)
N1—C6	1.366 (3)	C3—H3	0.93
C5—C9	1.419 (3)	C10—C7	1.506 (3)
C5—C6	1.432 (3)	C10—H10A	0.96
C5—C4	1.435 (3)	C10—H10B	0.96
C8—C9	1.356 (3)	C10—H10C	0.96
C8—C7	1.399 (3)	C4—C11	1.512 (3)
C8—H8	0.93	C11—H11A	0.96
C1—C2	1.358 (3)	C11—H11B	0.96
C1—C6	1.410 (3)	C11—H11C	0.96
C1—H1	0.93	C2—H2	0.93
			
C7—N1—C6	118.4 (2)	C7—C10—H10B	109.5
C9—C5—C6	114.0 (2)	H10A—C10—H10B	109.5
C9—C5—C4	127.6 (2)	C7—C10—H10C	109.5
C6—C5—C4	118.4 (2)	H10A—C10—H10C	109.5
C9—C8—C7	120.1 (2)	H10B—C10—H10C	109.5
C9—C8—H8	120.0	C3—C4—C5	118.0 (2)
C7—C8—H8	120.0	C3—C4—C11	117.5 (2)
C8—C9—C5	121.2 (2)	C5—C4—C11	124.4 (2)
C8—C9—Cl1	115.32 (18)	N1—C7—C8	122.2 (2)
C5—C9—Cl1	123.49 (18)	N1—C7—C10	117.8 (2)
C2—C1—C6	120.6 (2)	C8—C7—C10	120.0 (2)
C2—C1—H1	119.7	C4—C11—H11A	109.5
C6—C1—H1	119.7	C4—C11—H11B	109.5
N1—C6—C1	116.0 (2)	H11A—C11—H11B	109.5
N1—C6—C5	124.1 (2)	C4—C11—H11C	109.5
C1—C6—C5	119.9 (2)	H11A—C11—H11C	109.5
C4—C3—C2	123.4 (2)	H11B—C11—H11C	109.5
C4—C3—H3	118.3	C1—C2—C3	119.6 (2)
C2—C3—H3	118.3	C1—C2—H2	120.2
C7—C10—H10A	109.5	C3—C2—H2	120.2
			
C7—C8—C9—C5	−0.3 (4)	C4—C5—C6—C1	0.8 (3)
C7—C8—C9—Cl1	180.0 (2)	C2—C3—C4—C5	−0.2 (4)
C6—C5—C9—C8	0.7 (3)	C2—C3—C4—C11	178.3 (3)
C4—C5—C9—C8	−179.8 (2)	C9—C5—C4—C3	−179.9 (2)
C6—C5—C9—Cl1	−179.65 (18)	C6—C5—C4—C3	−0.4 (3)
C4—C5—C9—Cl1	−0.1 (4)	C9—C5—C4—C11	1.7 (4)
C7—N1—C6—C1	179.4 (2)	C6—C5—C4—C11	−178.8 (2)
C7—N1—C6—C5	0.4 (3)	C6—N1—C7—C8	0.1 (4)
C2—C1—C6—N1	−179.6 (2)	C6—N1—C7—C10	180.0 (2)
C2—C1—C6—C5	−0.5 (4)	C9—C8—C7—N1	−0.1 (4)
C9—C5—C6—N1	−0.7 (3)	C9—C8—C7—C10	−180.0 (2)
C4—C5—C6—N1	179.7 (2)	C6—C1—C2—C3	−0.1 (4)
C9—C5—C6—C1	−179.7 (2)	C4—C3—C2—C1	0.5 (4)
